# Spatial lipidomics reveals altered lipid profiles in *TMEM63A* mutant rats with hypomyelination

**DOI:** 10.1038/s41598-025-25371-z

**Published:** 2025-11-21

**Authors:** Junyu Wang, Liang Wang, Yu Zhang, Kai Gao, Jiangxi Xiao, Lin Nie, Jihang Luo, Shiqi Yang, Ye Wu, Yuwu Jiang, Huifang Yan, Jingmin Wang

**Affiliations:** 1 Children’s Medical Center, Peking University First Hospital, No.5 Le Yuan Road, Daxing District, Beijing, 102627 China; 2 Guidon (Beijing) Pharmaceutics LTD, Beijing, 100176 China; 3 Department of Radiology, Peking University First Hospital, Beijing, 100034 China; 4 Laboratory of Electron Microscopy, Ultrastructural Pathology Center, Peking University First Hospital, Beijing, 100034 China; 5 Beijing Key Laboratory of Molecular Diagnosis and Study On Pediatric Genetic Diseases, Beijing, 100034 China; 6 Key Laboratory for Neuroscience, Ministry of Education/National Health and Family Planning Commission, Peking University, Beijing, 100000 China; 7 Joint International Research Center of Translational and Clinical Research, Beijing, 100191 China

**Keywords:** *TMEM63A* mutation, Hypomyelination, Spatial lipidomics, MALDI-MSI, Genetics, Clinical genetics, Neuroscience, Development of the nervous system, Diseases of the nervous system, Genetics of the nervous system, Molecular neuroscience

## Abstract

Hypomyelinating leukodystrophies (HLDs) are genetic disorders characterized by deficient myelination. While *TMEM63A* variants are associated with HLD19, the specific lipid alterations in affected brain regions remain to be fully characterized. This study aimed to investigate the spatial distribution of lipid changes in a *Tmem63a* mutant rat model of hypomyelination. A homozygous *Tmem63a* c.500G > A p.(G167E) knock-in rat model (*Tmem63a*^*G167E/G167E*^) was established. Brain sections from *Tmem63a*^*G167E/G167E*^ and *Tmem63a*^*WT*^ rats (*n* = 3/group) were analyzed using MALDI-MSI for lipid profiling across nine distinct brain regions. Myelin structure was characterized by transmission electron microscopy (TEM) and g-ratio quantification. Statistical analyses included Mann-Whitney U tests for g-ratio distributions and ROC analysis for feature screening. Out of 702 analyzed features, 124 were differentially expressed. Lipids constituted the most altered class (43 features), including 22 glycerophospholipid, 9 fatty acid, 5 sphingolipid, 5 sterol lipid, and 2 prenol lipid species. These alterations were predominantly observed in white matter-rich regions and gray-white matter junctions. TEM revealed thinner and less dense myelin sheaths in *Tmem63a*^*G167E/G167E*^ rats, with a reduced proportion of optimal g-ratios. This study provides a comprehensive spatial lipidomic characterization in a *Tmem63a* mutant rat model, revealing significant lipid alterations associated with hypomyelination. These findings offer new insights into the pathology of hypomyelination and highlight specific lipid species for future investigation.

**Supplementary Information:**

The online version contains supplementary material available at 10.1038/s41598-025-25371-z.

## Introduction

Hypomyelinating leukodystrophies (HLDs) are a group of disorders characterized by developmental delay and hypomyelination in brain magnetic resonance imaging (MRI)^1^. Currently, 28 subtypes of HLDs have been defined in the Online Mendelian Inheritance in Man (OMIM) database (www.omim.org/). A critical feature of HLDs on brain MRI is hypomyelination, which presents as a T2-hyperintense signal in white matter and suggests a myelin deficit^2^. In addition to hypomyelination, demyelination and myelin vacuolization represent two other forms of myelin disorders^3,4^. Myelin, a concentric lipid-rich lamellae structure in the central nervous system (CNS), plays an essential role in speeding up action potential propagation and nourishing the axons it wraps^3^. Lipids account for a substantial portion (70–85%) of the dry weight of myelin^5^. Thus, lipids may thus represent sensitive markers of myelin status in physiological and pathological contexts. Given that lipids are a key component of myelin, understanding lipid alterations in myelin disorders remains an area requiring further investigation.

Metabolomics, particularly lipidomics, offers a powerful approach for acquiring quantitative and qualitative information on metabolic dynamics, which can provide insights into disease pathology or genetic alterations^6^. However, conventional metabolomics has inherent limitations in providing information about the spatial distribution of metabolites. In contrast, matrix-assisted laser desorption ionization mass spectrometry imaging (MALDI-MSI) is a relatively advanced technique that enables direct, label-free measurement of metabolites within tissues, thereby offering crucial spatial distribution information^7^. This approach has been successfully applied to study conditions such as dry eye disease^8^. To date, detailed lipids metabolic profiling of the brain, specifically the spatial distribution and quantitative changes of metabolites in specific brain regions as facilitated by MALDI-MSI, has not been widely reported in the context of HLDs.

The transmembrane protein 63 A (TMEM63A) gene was identified by our group and collaborators in 2019 as a novel gene associated with HLDs^9^. *TMEM63A* variants have been identified in patients presenting with intellectual disability/developmental delay (ID/DD) and hypomyelination, which was subsequently named hypomyelinating leukodystrophy type 19 (HLD19, OMIM 618688). To date, eleven patients with HLD19 have been reported globally, with dominant clinical characteristics including ID/DD, nystagmus, hypotonia, and T2-hyperintense signals in the white matter^9,10^. The presence of abnormal white matter signals in the brain MRI of patients with *TMEM63A* variants strongly suggests a disruption in myelination. To further characterize HLD associated with the *TMEM63A* c.500G > A p.(G167E) variant, we developed a *Tmem63a* knock-in rat model mirroring the patient-derived mutation. In this study, MALDI-MSI was applied to investigate the effect of *Tmem63a* mutation on lipids metabolism across different specific brain regions of *Tmem63a* mutant rats. The potential involvement of altered lipid metabolic pathways in HLD was also explored. Additionally, a novel semi-automated analysis software MyelTracer, was utilized for quantifying myelin structure as observed through transmission electron microscopy (TEM), to characterize the heterogeneous myelin sheaths in *Tmem63a* mutant rats. These findings offer fresh perspectives on the pathology of HLD.

## Materials and methods

This study adheres to the recommendations of international guidelines for the care and use of laboratory animals and was approved by the Animals Ethical Committee of Peking University First Hospital. All methods are reported in accordance with ARRIVE guidelines (https://arriveguidelines.org) for the reporting of animal experiments.

### Experimental animals and grouping

Sprague-Dawley (SD) rats were selected as the genetic background for animal model construction. A knock-in rat harboring the *Tmem63a* c.500G > A p.(G167E) mutation (*Tmem63a*^*G167E/G167E*^), originally identified in a human patient (*TMEM63A* c.503G > A p.(G167E)), was generated using CRISPR-Cas9 genome editing system. Model generation was commissioned to Beijing Vitalstar Biotechnology Co., LTD. [SCXK (Beijing) 2019-0002].

DNA samples, extracted from rat tails, were subjected to Sanger sequencing to determine genotypes. Based on genotyping, rats were allocated into two groups, *Tmem63a*^*G167E/G167E*^ and *Tmem63a*^*WT*^ (controls), respectively, with three male rats of the same three weeks of age in each group. All the rats were housed under specific pathogen-free (SPF) conditions at a constant room temperature of 22 ± 1 °C and approximately 60% ambient humidity, simulating a standard day-night system. Experimental male rats, weighing 200–250 g (3 weeks of age), were anesthetized via intraperitoneal injection of sodium pentobarbital at a dosage of 40 mg/kg.

### Tissue sectioning and matrix application

Fresh-frozen brain samples were cryosectioned at 12 μm thickness using a cryomicrotome at -20 °C (Leica CM1950; Leica Microsystems, Wetzlar, Germany). Sections were transferred onto pre-cooled ITO conductive slides, which were labeled with a Teach Marker and scanned at 7200 dpi resolution.

Matrix spraying was performed using an electric field-assisted sprayer. The matrix solution consisted of 7.9 mg of 1,5-Diaminonaphthalene (1,5-DAN) hydrochloride derivative, prepared by sequentially adding 100µL of 1 mol/L hydrochloride and 800µL of distilled water, followed by 900µL of ethanol. Spraying parameters were set as follows: pressure, 0.3 MPa; flow rate, 20 µL/min; temperature, 80 °C; and matrix spray volume: 0.1 mg/cm^2^.

### MALDI-MSI experiment

Mass spectrometry imaging was performed using the rapifleX MALDI-TOF/TOF MS (Bruker Daltonics, Bremen, Germany) equipped with a 10 kHz smartbeam 3D laser. Parameters were set as follows: negative ion mode; laser power, 60%; lens voltage, 11.3 kV; reflector voltage, 20.8 kV, with a mass-to-charge ratio (m/z) ranging from 100 to 1200 Da. The imaging spatial resolution was 100 μm. Rats brain tissues were mounted onto two slides for subsequent experiments. The matrix for both slides was sprayed in the same batch, thereby ensuring minimal batch effects. Data from the two slides were collectively processed using total ion current (TIC) normalization, ensuring negligible matrix spray variation.

Raw data were read, peaks recognized, and mass spectra obtained using the digital imaging software flexImaging 5.0 (Bruker Daltonics). MS imaging data analysis, including baseline subtraction, signal smoothing, cluster analysis, and PCA analysis, was conducted using SCiLS Lab 2022 Pro (Bruker Daltonics).

### Hematoxylin-eosin (HE) staining

Hematoxylin-eosin (HE) staining was performed on the same tissue sections used for mass spectrometry imaging. Sections were briefly immersed in 70% ethanol for 2 min to remove the matrix, followed by routine hematoxylin and eosin staining. Stained sections were then observed and photographed under a microscope for the division of brain regions.

### Metabolic pathway analysis and metabolites identity

To identify statistically significant lipid-related metabolic pathways, the 124 differentially expressed features were analyzed using the MetaboAnalyst web platform (https://www.metaboanalyst.ca/). Pathways exhibiting significant enrichment were defined by a p-value less than 0.05 (corresponding to a -log10(0.05) value = 1.3), with a stricter threshold of *p* < 0.001 (equivalent to a -log10(0.001) value = 3) indicating high significance. Subsequently, relevant lipid metabolic pathways were retrieved and cross-referenced with the Kyoto Encyclopedia of Genes and Genomes (KEGG) database^11^ (https://www.genome.jp/kegg/).

Glycerophospholipid metabolism and sphingolipid metabolism were specifically analyzed. The glycerophospholipids analyzed included phosphatidylcholine (PC), phosphatidylethanolamine (PE), phosphatidylserine (PS), phosphatidic acid (PA), lysophosphatidylcholine (LPC), lysophosphatidylethanolamine (LPE) and lysophosphatidic acid (LPA). Additionally, sphingolipids such as sphingosine (Sph), sphingomyelin (SM), glucosylceramide (GlcCer), lactosylceramide (LacCer) and sulfogalactosylceramide (SGCer) were invesgated. These aforementioned lipid-related metabolites were also observed and identified.

### The transmission electron microscopy (TEM) and analysis of myelin structure

Following euthanasia via deep anesthesia, rats from both *Tmem63a*^*G167E/G167E*^ and *Tmem63a*^*WT*^ groups were perfused by PBS, followed by 2.5% glutaraldehyde for 60 min (1mL/min). The corpus callosum was then dissected into pieces of 1 × 1 × 1mm^3^ and immersed into glutaraldehyde for overnight fixation at 4℃. Dissected tissue underwent three PBS washes before osmium tetroxide treatment. After another PBS wash, tissues were dehydrated through an ascending ethanol series and embedded in Epon. Ultrathin sections were then cut, collected on naked grids, and stained with uranyl acetate and lead citrate.

Grids were examined using an FEI Tecnai G^2^ 20 Twin electron microscope operating at an acceleration voltage of 80 kV. Images were captured with a CD camera (Gatan 832) from 3 male rats per genotype (6 rats total, three weeks of age), utilizing the aforementioned parameters. Three non-overlapping fields of view were randomly selected from the recorded images of each rat. Myelin structures were delineated and quantified by MyelTracer, a novel stand-alone software for semi-automated g-ratio quantification, which is based on the Open Computer Vision Library (OpenCV) and developed by the Massachusetts Institute of Technology^12^.

### Statistical analysis

Data were analyzed using GraphPad Prism (version 9.0.0, GraphPad Software, San Diego, CA, USA) and SCiLS Lab 2022 Pro (Bruker Daltonics). For comparison between two groups, two-tailed unpaired Student’s t-tests were performed. G-ratio distributions were analyzed using Mann-Whitney U tests. Receiver operating characteristic (ROC) analysis was performed to differentially expressed peaks, with an area under the curve (AUC) greater than 0.8 or less than 0.2 indicating statistical significance (*p* < 0.05). *P* < 0.05 was considered statistically significant.

## Results

### Establishment of the mutant *Tmem63a* rat models

**Fig. 1:**
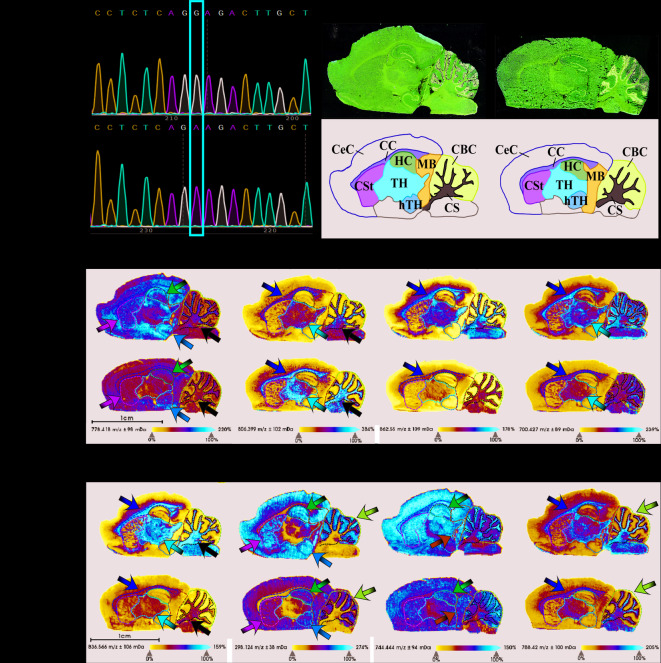
Characterization of the *Tmem63a*^*G167E/G167E*^ rat model and spatial lipid alterations in brain regions. (**a**) Sanger sequencing of *Tmem63A*^*G167E/G167E*^ and *Tmem63a*^*WT*^ rats. (**b**) HE staining of *Tmem63a*^*G167E/G167E*^ and *Tmem63a*^*WT*^ rats, illustrating the nine analyzed brain regions. Brain regions: CS, cerebellum stem; CC, corpus callosum; TH, thalamus; CSt, corpus striatum; hTH, hypothalamus; CeC, cerebral cortex; CBC, cerebellar cortex; HC, hippocampus and MB, midbrain. (**c**), (**h**), (**i**), (**j**) Representative MALDI-MSI images showing reduced expression of PC (e.g., PC(15:0/18:2)), Sph, PE (e.g., PE(18:0/18:1)), and PS (e.g., PS(18:0/18:1)) in gray-white matter junctions and gray matter-rich regions of *Tmem63a*^*G167E/G167E*^ rats. (**d**) The MALDI-MSI image showing increased expression of PC (e.g., PC(15:0/20:2)) in white matter-rich regions of *Tmem63a*^*G167E/G167E*^ rats. (**e**), (**f**) and (**g**) The MALDI-MSI images showing reduced expression of PC (e.g., PC(20:4/20:5)), PE (e.g., PE(P-16:0/18:1)), and glucosylceramide (GlcCer) (e.g., GlcCer(d18:1/26:1)) in white matter-rich regions of *Tmem63a*^*G167E/G167E*^ rats. Color-coded arrows: yellow, the corpus callosum; white, the cerebellum stem; red, the thalamus; green, the corpus striatum; orange, the hypothalamus; pink, the hippocampus; blue, the midbrain; purple, the cerebellar cortex. Scale bar=1 mm (n = 3).

The mutant rat model carrying the homozygous *Tmem63a* c.500G > A p.(G167E) mutation was successful established. This genotype was confirmed by Sanger sequencing of genomic DNA in the *Tmem63a*^*G167E/G167E*^ group (Fig. 1a).

### Overview of lipid related features in nine brain regions

In this study, a total of 702 features were analyzed through MALDI-MSI experiments, among which 124 features showed statistically significant changes (Supplemental Table 1). Based on their chemical properties, these 124 differentially expressed features were classified into seven categories: lipids (43 features), amino acids and derivatives (20 features), organic acids (20 features), nucleotides and nucleosides (18 features), carbohydrates & sugar phosphates (8 features), vitamins & Co-factors (1 feature), and other small molecules (14 features) not falling into the preceding six categories. Further, based on their lipid structure and properties, the 43 lipid features were subclassified into glycerophospholipids (22 features), fatty Acids (9 features), sphingolipids (5 features), steroids (5 features), and prenol lipids (2 features). Given their abundance in myelin, glycerophospholipids and sphingolipids were subjected to detailed analysis across the nine brain regions (Table 1).

**Table 1:** 27 species glycerophospholipids and sphingolipids features and their alteration in *Tmem63A*^*G167E/G167E*^ rats.

LCMS	white matter-rich regions			gray-white matter junctions		gray matter-rich regions			
	CS	CC	TH	CSt	hTH	CeC	CBC	HC	MB
PC(15:0/18:2)	-	-		-	-			-	-
PC(18:2/16:1)				-	-	-		-	
PC(15:0/20:2)	+	+	+						
PC(18:0/18:1)		+							
PC(18:0/20:3)	-	-	-	-					
PC(20:4/20:5)	-	-	-	-					
PC(20:0/20:3)	-	-	-						
PC(20:0/22:0)		+							
LPC(O-18:0/0:0)				-					
PE(P-16:0/18:1)	-	-							
PE(14:0/18:0)	-	-	-						
PE(15:0/20:2)	-	-	-			-			
PE(18:0/18:1)			-	-					-
PE(P-18:0/20:1)	-	-	-						
PE(16:0/22:4)	-	-							-
LPE(0:0/16:1(9Z))				+		+			
LPE 20:4									-
PS(18:0/18:1)	-	-					-		
PS(18:0/22:6)	-	-							
PA(16:0/16:0)	-	-		-					
LPA(13:0)		-		-	-			-	-
GPC	+	+	+				+	+	+
Sphingosine				-	-		-	-	
GlcCer(d18:1/26:1)	-	-	-						
GlcCer(d18:1/24:1)	-	-	-	-					
LacCer(d18:1/16:0)	-	-	-	-					
SGCer(d18:1/14:0)		-		-	-			-	-

+, increase; -, decrease; CS, cerebellum stem; CC, corpus callosum; TH, thalamus; CSt, corpus striatum; hTH, hypothalamus; CeC, cerebral cortex; CBC, cerebellar cortex; HC, hippocampus; MB, midbrain.

Nine brain regions were accurately classified with the aid of HE staining, providing a foundation for the spatial localization of lipid features in the brain. Based on white matter content, the nine regions were categorized into white matter-rich regions (the cerebellum stem, corpus callosum, and thalamus), gray-white matter junctions (the corpus striatum and hypothalamus), and gray matter-rich regions (the hippocampus, midbrain, cerebellar cortex, and cerebral cortex) (Fig. 1b). In the *Tmem63a*^*G167E/G167E*^ group, the expression of 17 lipid species of glycerophospholipids was visually decreased, while the remaining 5 lipid species showed an increase. Concurrently, all 5 sphingolipid species were decreased.

#### Alterations of lipid features in white matter-rich regions

Both glycerophospholipids and sphingolipids are predominantly distributed in the white matter-rich regions. We utilized MALDI-MSI to illustrate the changes in both glycerophospholipids and sphingolipids metabolism within these regions. These areas were also stained with HE for spatial comparison with MALDI-MSI findings in white matter-rich regions.

Quantitative differences in lipid features were evident in the cerebellum stem between groups, with five lipid species of PC and PE, two of PS and one of PA and LPA altered in the *Tmem63a*^*G167E/G167E*^ group. Integrated glycerophospholipids analysis showed a general decrease in PC, PE, PS and PA (white arrow in Fig. 1c), except for an increase in PC(15:0/20:2) (white arrow in Fig. 1d). Additionally, ceramide-related features decreased, and the GlcCer to LacCer ratio was altered (white arrow in Fig. 1g).

Agenesis of the corpus callosum is associated with various diseases of the nervous system, including HLD. In our study, similar to the changes observed in the cerebellum stem, the *Tmem63a*^*G167E/G167E*^ rats exhibited decreased levels of PE, PS, PA and ceramide-related features were decreased in the corpus callosum compared to controls (yellow arrow in Fig. 1e,f,g and j). Conversely, different lipid species of PC showed an opposite trend in this region (yellow arrow in Fig. 1d).

Contrary to observations in the cerebellum stem and corpus callosum, PS expression in the thalamus shows no significant differences between the two groups. However, an increased expression of PC(15:0/20:2) was observed in the *Tmem63a*^*G167E/G167E*^ group compared to controls (red arrow in Fig. 1d). Similar to trends in the cerebellum stem and corpus callosum, various lipid species of PC and all lipid species of PE were decreased in the *Tmem63a*^*G167E/G167E*^ group (red arrow in Fig. 1f). Additionally, three lipid species of ceramide-related features were reduced in the thalamus (red arrow in Fig. 1g).

These findings highlight that glycerophospholipids and ceramide-related features are primarily distributed in white matter-rich regions and undergo significant decreases in expression in the *Tmem63a*^*G167E/G167E*^ group.

#### Alterations of lipid features in gray-white matter junctions

In the gray-white matter junctions, the corpus striatum of the *Tmem63a*^*G167E/G167E*^ group showed significantly reduced levels and number of lipid species for PC, LPC, PE, PA and LPA (green arrow in Fig. 1c). However, no obvious changes were observed in PS between the two groups. Additionally, Sph and three lipid species of ceramide-related features were decreased in the *Tmem63a*^*G167E/G167E*^ group (green arrow in Fig. 1h).

In the hypothalamus, two lipid species of PC (PC(15:0/18:2) and PC(18:2/16:1)) (orange arrow in Fig. 1c), LPA(13:0), Sph and SGCer(d18:1/14:0) decreased in *Tmem63a*^*G167E/G167E*^ group (orange arrow in Fig. 1h).

#### Alterations of lipid features in gray matter-rich regions

Compared with the white matter-rich regions and gray-white matter junctions, the number of altered glycerophospholipids notably lower in gray matter-rich regions. The expression of two lipid species of PC and PE and a species of LPA was reduced in the *Tmem63a*^*G167E/G167E*^ group.

In the cerebellar cortex, the expression of PS(18:0/18:1) and Sph visibly decreased in the *Tmem63a*^*G167E/G167E*^ group (purple arrow in Fig. 1j and h). The expression of PC(15:0/18:2), PC(18:2/16:1), and Sph decreased in the *Tmem63a*^*G167E/G167E*^ group in the hippocampus (pink arrow in Fig. 1j and h).

In the midbrain, PC(15:0/18:2), PE(18:0/18:1), PE(16:0/22:4) and LPA(13:0) were down-regulated in the *Tmem63a*^*G167E/G167E*^ group (blue arrow in Fig. 1i).

### Alterations of glycerophospholipid and sphingolipid metabolic pathways

**Fig. 2:**
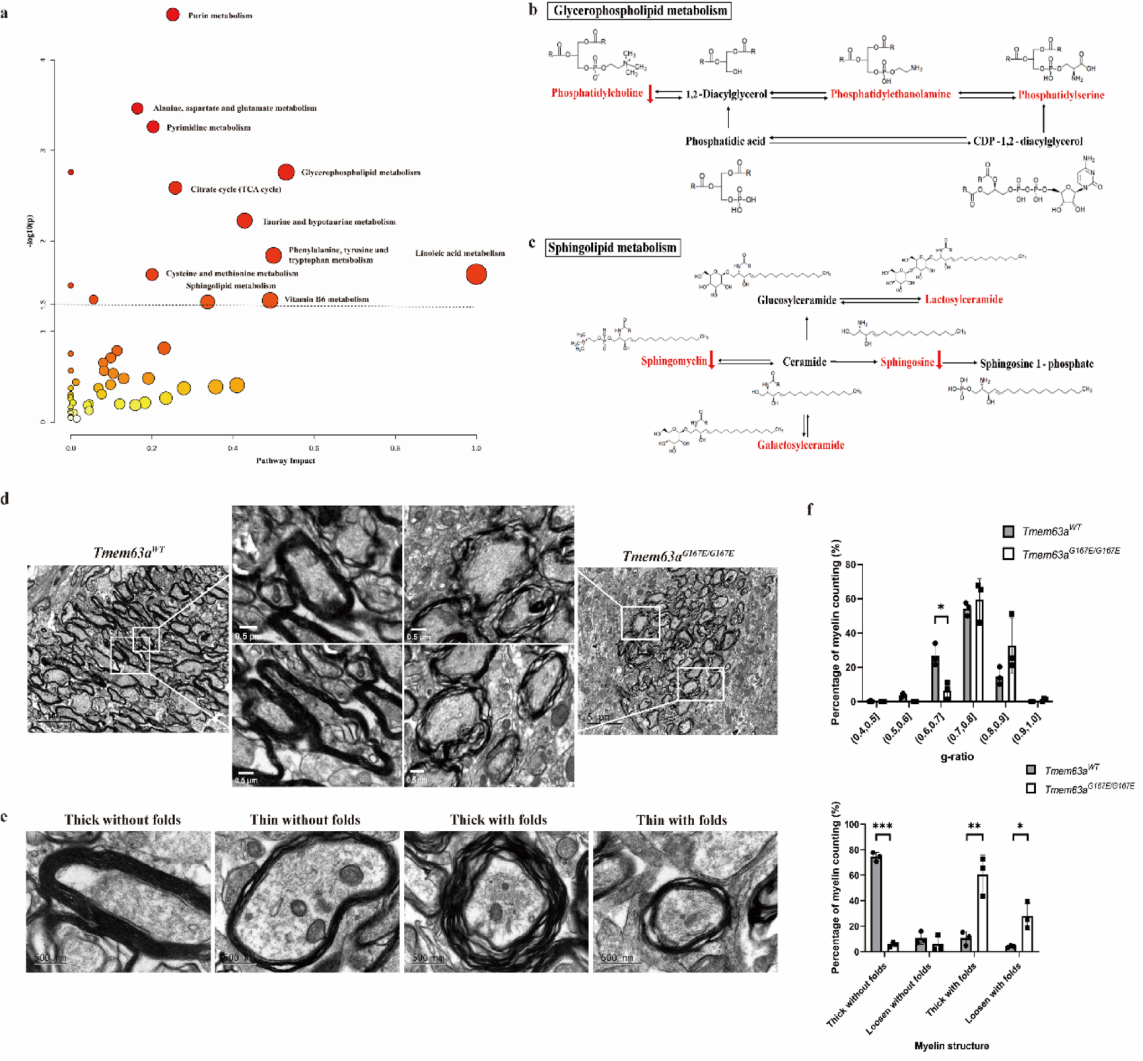
Metabolic pathway enrichment and myelin ultrastructural characterization. (**a**) Enriched metabolic pathways for the 124 differentially expressed features. (**b**) Glycerophospholipid metabolism pathway. (**c**) Sphingolipid metabolism pathway. (**d**) TME image of myelin structure in *Tmem63a*^*G167E/G167E*^ and *Tmem63a*^*WT*^ rats (Scale bar=5μm and 0.5μm). (**e**) Four types of myelin structures observed in *Tmem63a*^*G167E/G167E*^ and *Tmem63a*^*WT*^ rats (Scale bar=500nm). (**f**) G-ratio distribution and proportion of four types of myelin structures in *Tmem63a*^*G167E/G167E*^ and *Tmem63a*^*WT*^ rats (n=3 rats per group).

Following the MALDI-MSI measurements, we identified the quantitative and qualitative metabolic profiles of the nine brain regions in both rat groups. The obviously altered lipid features were mapped onto the glycerophospholipid metabolism and sphingolipid metabolism pathways (Fig. 2a). Both the glycerophospholipid metabolism pathway and sphingolipid metabolism pathways exhibited large pathway impact values, indicating a greater biological influence on these pathways and suggesting that the metabolites involved are more critical. Concurrently, the -log10(p) values for both pathways were ≥ 1.3, denoting their enrichment as highly statistically significant and less likely to be attributable to random chance.

#### Alterations of glycerophospholipid metabolism pathway

Changes in various lipid-related features within the glycerophospholipid metabolism pathway were found across all nine brain regions (Fig. 2b). These alterations were particularly prominent in the white matter-rich regions and gray-white matter junctions, where the expression levels of various glycerophospholipids and their lipid species were affected. All four lipid subclasses of glycerophospholipids (PC, PE, PS and PA) with significant alterations were expressed in the cerebellum stem and corpus callosum. Beyond the cerebral cortex and two aforementioned brain regions, only PC and PS alterations were noted in other brain regions, with only PS changes occurring in the cerebral cortex. Overall, PC, PS, and PE, which are three major lipid species within the glycerophospholipid metabolism pathway, exhibited a down-regulated trend in the *Tmem63a*^*G167E/G167E*^ group.

#### Alterations of sphingolipid metabolism pathway

Sphingolipid metabolism was identified as another altered metabolic pathway associated with *Tmem63a* mutation, primarily involving Sph and ceramide-related features as the dominant substances (Fig. 2c). Similar to glycerophospholipids, ceramide-related features predominantly showed alterations in the white matter-rich regions and gray-white matter junctions. Changes in Sph were clustered in the gray-white matter junctions and gray matter-rich regions. Both ceramide-related features and Sph were decreased in the *Tmem63a*^*G167E/G167E*^ group.

### The myelin structure of *Tmem63a*^*G167E/G167E*^ and *Tmem63a*^*WT*^ rats

To further investigate whether rats exhibited ultrastructural changes in myelin analogous to the macroscopic lipid alterations observed, we performed TEM on the myelin structure of both *Tmem63a*^*G167E/G167E*^ and *Tmem63a*^*WT*^ rats. In contrast to *Tmem63a*^*WT*^ rats, the myelin sheaths in the *Tmem63a*^*G167E/G167E*^ group appeared less dense (Fig. 2d). The proportion of myelin in the *Tmem63a*^*WT*^ group with a g-ratio between 0.6 and 0.7 was significantly higher than that in *Tmem63a*^*G167E/G167E*^ group (*p* < 0.05), indicating a greater prevalence of thicker myelin sheaths in *Tmem63a*^*WT*^ rats (Fig. 2f).

Myelin sheaths were categorized into four types of: ‘Thick without folds’, ‘Thin without folds’, ‘Thick with folds’ and ‘Thin with folds’ (Fig. 2e). ‘Thick’ myelin sheaths were defined as having a g-ratio < 0.8, while ‘without folds’ indicated the presence of two or fewer folds within the myelin sheaths. The percentage of ‘thick without folds’ myelin type was obviously higher in *Tmem63a*^*WT*^ rats than in *Tmem63a*^*G167E/G167E*^ rats (*p* < 0.001). Conversely, the percentages of ‘thick with folds’ and ‘thin with folds’ myelin types were higher in *Tmem63a*^*G167E/G167E*^ rats compared to *Tmem63a*^*WT*^ rats, with p < 0.01 and p < 0.05, respectively (Fig. 2f). Overall, the myelin in *Tmem63a*^*WT*^ rats exhibited greater thickness and density than that in *Tmem63a*^*G167E/G167E*^ rats, suggesting an association between the *Tmem63a* mutation and reduced myelin density and thickness in rats.

## Discussion

Given the significant challenges in obtaining brain tissue samples from patients, and considering that rats serve as important models for disease research owing to the high similarity of their genome to the human genome (http://rgd.mcw.edu), this study first successfully established the *Tmem63a* knock-in rat model to explore the pathophysiological characteristics of hypomyelination. The selection of rat age for this study was guided by the characteristic clinical phenotype of HLD19. HLD19 patients commonly present with phenotypes such as developmental delay and nystagmus within the first few months postnatally, with some individuals showing alleviation of clinical manifestations as early as around 4 years of age^9^. Three-week-old rats approximate the human developmental stage of approximately 2 years of age^13^, a period when HLD19 patients typically exhibit their most prominent phenotypes. Therefore, investigating brain lipid metabolism and myelin structure at this specific developmental age in rats is particularly informative.

Lipids constitute a crucial component of myelin. Notably, myelin’s lipid composition, characterized by a distinctive 2:2:1 ratio of cholesterol: phospholipids: glycolipids^14^, differs from that of ordinary eukaryotic cell membranes. The application of MALDI-MSI to investigate lipid metabolism in hypomyelination models can offer novel avenues for the pathological study of hypomyelination. Glycerophospholipids constitute a major and abundant component of phospholipids in cell membranes. The major glycerophospholipids in myelin include PC, PE, and PS, which are also direct components of eukaryotic cell membranes^15^, with slight variations in myelin depending on the mammal species and age. Myelination involves the rapid and extensive synthesis and expansion of oligodendrocyte plasma membranes. PC is the most abundant glycerophospholipid in both cell and myelin membranes, typically comprising 41–57 mol%, with most PC localizing to the outer leaflet. An adequate supply of PC is essential for myelin formation, contributing to the structural stability and membrane fluidity^16^. PC appears to influence myelination through various indirect pathways. Impaired synthesis or reduced levels of PC and its key component, choline, may directly limit the generation of new membranes and the efficient expansion of myelin layers^17^. Choline transporters, such as SLC44A1 and SLC44A5, are crucial for oligodendrocyte differentiation and myelination. Specifically, the absence of SLC44A1 can lead to lipid metabolism dysregulation, particularly affecting the synthesis of plasmalogens essential for myelin biogenesis, thereby impairing oligodendrocyte differentiation and myelin formation^17^. Concurrently, the myelination process demands substantial amounts of protein and lipid synthesis, which places a significant load on the secretory pathway of the endoplasmic reticulum (ER). Indeed, the blockage of PC production is known to cause ER stress and activate the ER-stress sensor, Ire1, to induce unfolded protein response (UPR)^18^. The occurrence of ER stress can lead to the accumulation of unfolded or misfolded proteins within the ER lumen, and negatively impact the expression and production of key myelin-associated proteins (eg. MBP, PLP), thereby affecting myelination^19^. The overall trend of decreased PC in the brain tissue of *Tmem63a* mutant rats in this study is also consistent with findings from previous research. Furthermore, during myelination, the interconversion between PC and PE, leading to a gradual decrease in the PC/PE ratio, is a hallmark of myelination. As the second most abundant phospholipid in cell membranes PE has a smaller headgroup that results in a more conical shape compared with PC. PE is involved in the negative curvature of the plasma membrane, which is associated with the compact structure of multilayered myelin membranes^20^. In the present study, the overall trend of decreased PE in the brain tissue of *Tmem63a* mutant rats, along with the observed vacuolar changes in myelin, is consistent with these findings. At the ultrastructural level, alterations in phospholipid composition have the potential to influence myelin properties through their impact on membrane curvature. The spontaneous curvature of PE plays a vital role in membrane bending and tubulation, processes that are crucial for fission and fusion steps in vesicular transport^21^. This may be crucial for oligodendrocytes to transport newly synthesized membrane proteins and lipids to myelin-forming sites. PS is predominantly localized to the inner membrane leaflet. Owing to its negatively charged headgroup, PS and its phosphorylated derivatives provide a cytoplasm platform for diverse enzymes activities, thereby facilitating signal transduction^16^. These signaling pathways may be crucial for oligodendrocyte proliferation, differentiation, and myelination. During efferocytosis, PS exposition on the ectoplasmic leaflet of apoptotic cells can also release an ‘eat me’ signal, facilitating recognition by macrophages^16^. Aberrant PS metabolism may affect the normal apoptotic process of oligodendrocytes or the clearance of their debris, thereby influencing myelin repair and remodeling.

Each lipid subclass (e.g., PC, PE) comprises numerous individual lipid species, which differ in fatty acid chain saturation and double bond position. Minor structural differences between positional isomers can markedly change the biological function of a lipid^22,23^. Research has shown that a more central location of double bonds within a lipid’s acyl chain can better promote membrane fluidity^22^. In our study, different lipid species within the same subclass exhibited varying expression trends. While some species might show an increase, the predominant trend for the majority of species within these subclasses was a decrease, leading to an overall reduction in these major lipid components. Crucially, the trend in the major structural lipid subclasses (glycerophospholipids and sphingolipids) is consistent with the observed thinner and less dense myelin sheaths in the *Tmem63a*^*G167E/G167E*^ rats. Myelination and stability rely on the balanced composition and sufficient quantity of these major lipid classes.

Sph, an intermediate metabolite between ceramide and sphingosine-1-phosphate (S1P), plays a pivotal role in sphingolipid metabolism. Sph is involved in the maturation of oligodendrocytes, survival, migration and the myelination process^24,25^. The inflammatory cytokines stimulate Sph increase inducing oligodendrocytes death and causing demyelination^26^. GlcCer is an important glycosphingolipid, serving as a precursor for many complex glycosphingolipids, including GalCer and LacCer. GlcCer and its derivatives promote axon-glial adhesion during myelinogenesis, axo-glial interactions at nodes of Ranvier, and myelin formation^27^. GlcCer has been shown to influence fundamental cellular behaviors such as proliferation, lipid homeostasis, and intracellular trafficking as well^28^. Consequently, alterations in GlcCer levels could potentially impact oligodendrocyte lineage progression and thereby contributing to the hypomyelination phenotype. In the present study, almost all alterations in glycerophospholipid and sphingolipid species were concentrated in white matter regions. In contrast, changes in Sph were primarily observed in gray-white matter junctions and even gray matter regions, and Sph expression did not show an increase in the brain tissue of *Tmem63a* mutant rats. The concentration of Sph alterations in these critical transitional zones suggests that oligodendrocyte function in these specific regions may be disrupted. Although myelination primarily occurs in white matter, these crucial transitional zones also play an important role in initiating or maintaining myelin formation. Previous studies have shown that gray matter abnormalities, such as epilepsy, can lead to reduced myelination in the corpus callosum, increased demyelination, and an increase in oligodendrocyte lineage cell death^29^. Glycerophospholipids and sphingolipids undergo dynamic changes during myelination^20^. The absence of a decrease or increase in Sph in white matter regions could be attributed to dynamic supplementation by other lipids or the dynamic conversion of Sph into other lipid species.

TEM has provided unparalleled access to the ultrastructural features of myelin sheaths. The ratio of axon diameter to total fiber diameter (known as g-ratio) served as a metric for assessing myelin thickness. This ratio typically ranges from 0.4 to 0.8 across species, with an optimal g-ratio of approximately 0.7 for efficient conduction in the central nervous system of rats^30^. Consequently, in our study, the g-ratio values predominantly fell within the range of (0.7, 0.8] in both *Tmem63a*^*G167E/G167E*^ and *Tmem63a*^*WT*^ rats, reflecting the developmental stage of myelination at three weeks of age. Motor function is intricately linked to human myelination. *Tmem63a*^*G167E/G167E*^ exhibited thinner myelin structures, specifically a reduced proportion of myelin with a g-ratio in the (0.6, 0.7] range compared to controls. This observation is consistent with the T2-hyperintense white matter signals observed in brain MRI of HLD19 patients, further supporting the relevance and validity of the *Tmem63a* rat model for HLD19.

To definitively investigate whether a causal relationship exists between altered lipid metabolism and hypomyelination, several further studies are warranted. A longitudinal time-course study in the *Tmem63a*^*G167E/G167E*^ rat model will be crucial, involving the examination of lipid profiles and myelin structure at earlier developmental time points (e.g., postnatal day 7 (1 week) and day 14 (2 weeks)). Additionally, lipid rescue experiments are crucial to investigation. In these studies, specific lipid species found to be significantly decreased in the mutant rats (e.g., PC or GlcCer) would be exogenously administered to assess their potential to ameliorate the hypomyelination phenotype. Such interventions could involve direct brain delivery or dietary supplementation, if applicable. Furthermore, the utilization of in vitro oligodendrocyte culture systems derived from our rat model is essential. These systems, coupled with advanced lipidomics and live-cell imaging, would allow us to explore the cellular mechanisms by which the *TMEM63A* mutation impacts lipid synthesis and trafficking, enabling us to track lipid dynamics. Collectively, these multifaceted approaches will provide more direct evidence to establish causality and identify potential therapeutic interventions.

## Conclusions

This study successfully established a *Tmem63a* knock-in rat model, providing a valuable platform for investigating hypomyelination. Through the application of spatial lipidomics (MALDI-MSI) and ultrastructural analysis (TEM), we comprehensively characterized region-specific lipid alterations and associated myelin structural changes in the mutant rats. Our findings reveal significant dysregulation across various lipid classes, particularly glycerophospholipids and sphingolipids, predominantly in white matter-rich regions and gray-white matter junctions. These observed lipid profiles are consistently associated with thinner and less dense myelin sheaths. This work offers novel insights into the lipid landscape of *TMEM63A*-associated hypomyelination, contributing to a deeper understanding of its pathology and laying a foundation for future targeted investigations into potential biological markers and intervention strategies.

## Supplementary Information

Below is the link to the electronic supplementary material.

Supplementary Material 1

Supplementary Material 2

Supplementary Material 3

Supplementary Material 4

## Data Availability

The datasets generated and/or analysed during the current study are available in the ClinVar repository, https://www.ncbi.nlm.nih.gov/clinvar/variation/689459/?oq=SCV006082227&m=NM_014698.3(TMEM63A):c.503G%3EA%20(p.Gly168Glu).

## Abbreviations

CBC: Cerebellar cortex
CC: Corpus callosum
CeC: Cerebral cortex
CNS: Central nervous system
CS: Cerebellum stem
CSt: Corpus striatum
DD: Developmental delay
GalCer: Galactolipids
GlcCer: Glucosylceramide
GPC: Glycerophosphocholine
HC: Hippocampus
HE: Hematoxylin–eosin staining
HLD: Hypomyelinating leukodystrophy
hTH: Hypothalamus
KEGG: Kyoto encyclopedia of genes and genomes
LacCer: Lactosylceramide
LPA: Lysophosphatidic acid
LPC: Lysophosphatidylcholine
LPE: Lysophosphatidylethanolamine
MALDI-MSI: Matrix-assisted laser desorption ionization mass spectrometry imaging
MB: Midbrain
MRI: Magnetic resonance imaging
KEGG: Kyoto encyclopedia of genes and genomes
PA: Phosphatidic acid
PC: Phosphatidylcholine
PE: Phosphatidylethanolamine
PS: Phosphatidylserine
RGD: Rat genome database
SD: Sprague–Dawley rats
SGCer: Sulfogalactosylceramide
SM: Sphingomyelin
SPF: Specific pathogen-free
Sph: Sphingosine
S1P: Sphingosine-1-phosphate
TEM: Transmission electron microscopy
TH: Thalamus
TMEM63A: Transmembrane protein 63A
