# Supplementary material for: Spatial lipidomics reveals altered lipid profiles in TMEM63A mutant rats with hypomyelination
Source: Sci Rep. 2025 Nov 21;15:41398. doi: 10.1038/s41598-025-25371-z (PMC12638909; doi:10.1038/s41598-025-25371-z)

**
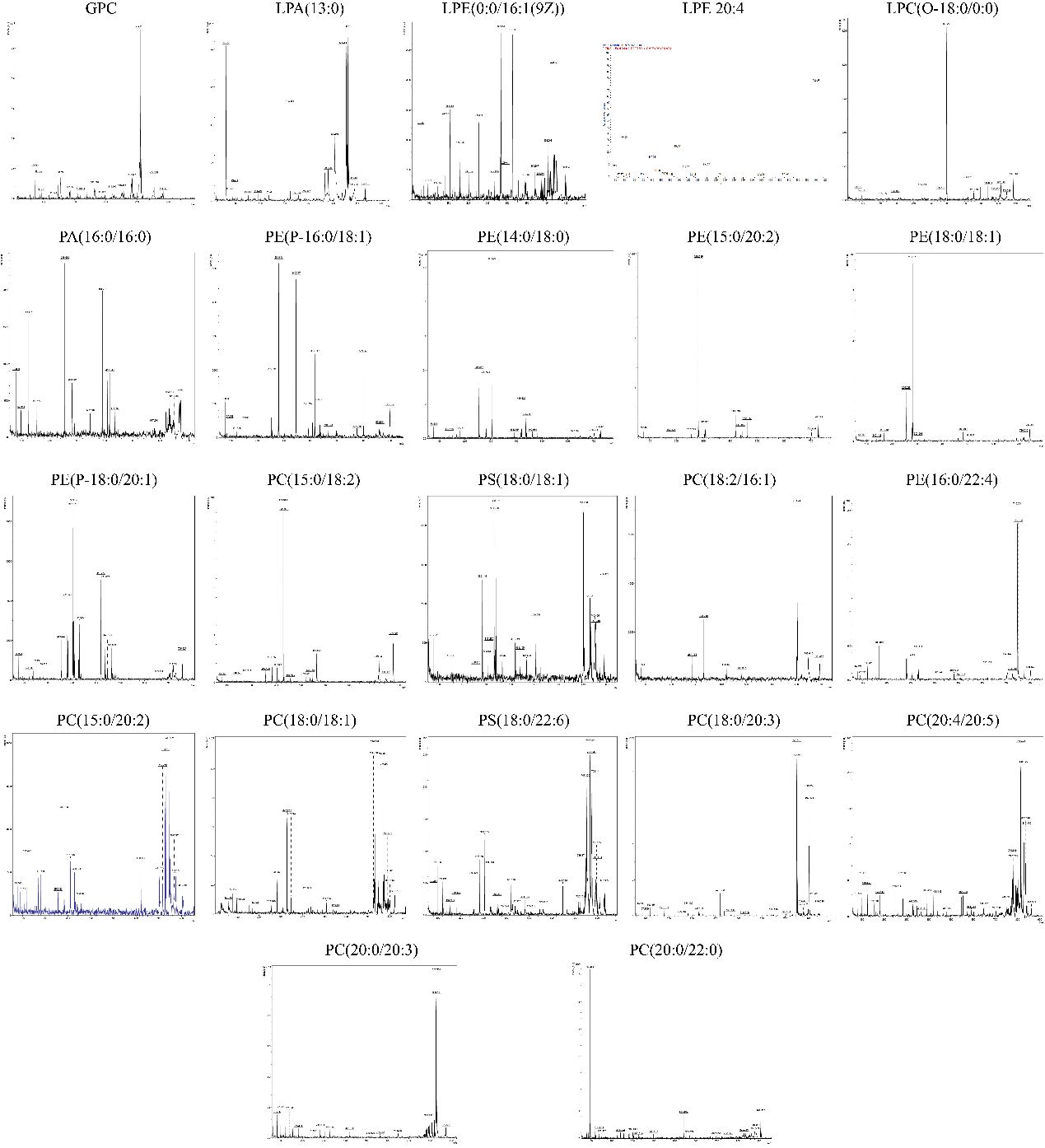
Supplemental Figure 1.** MALDI qualitative analysis figures for the 22 glycerophospholipids.

**Supplemental Figure 2.** MALDI qualitative analysis figures for the 5 Sphingolipids.


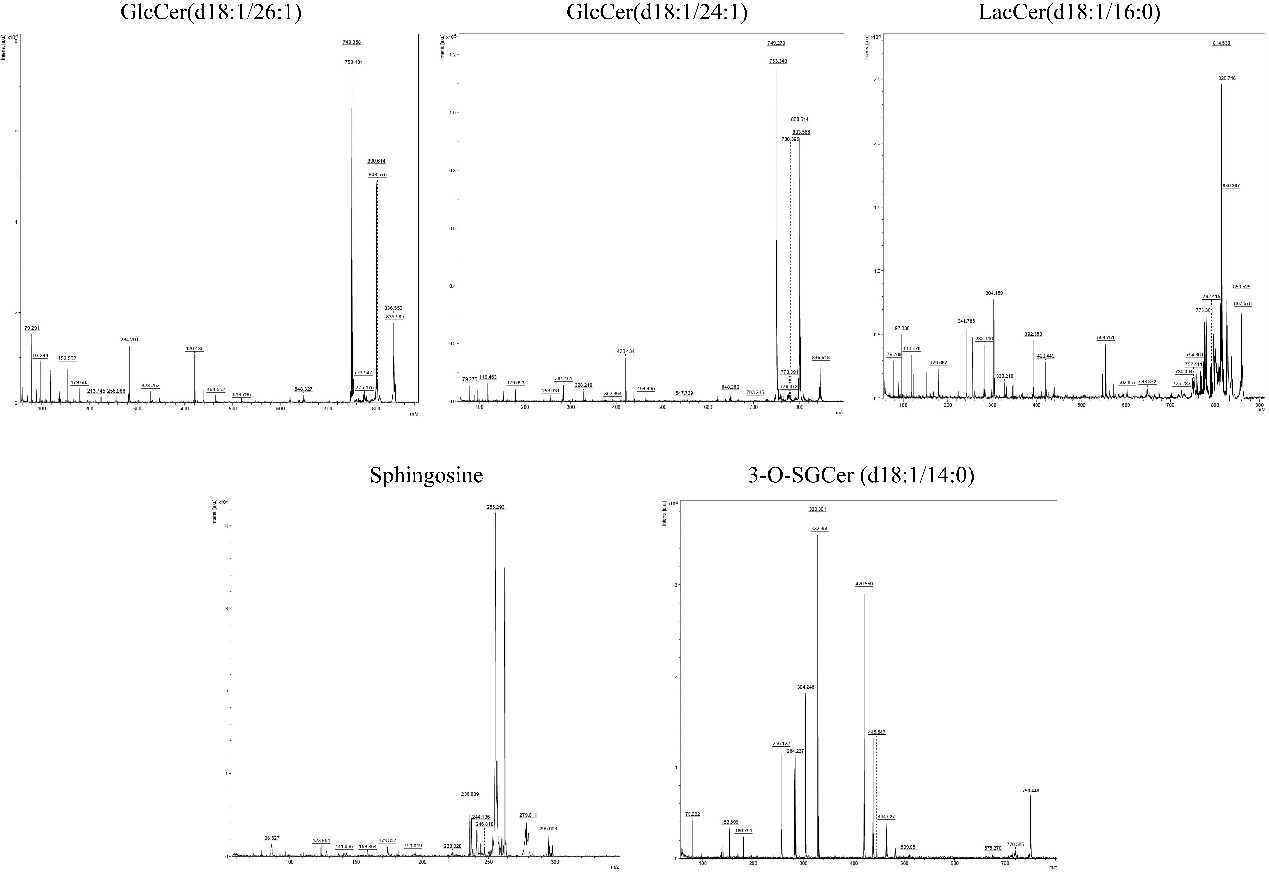

Supplement: Supplementary file 1 — Supplementary Material 1 [file 41598_2025_25371_MOESM1_ESM.docx]
